# Functioning of PPR Proteins in Organelle RNA Metabolism and Chloroplast Biogenesis

**DOI:** 10.3389/fpls.2021.627501

**Published:** 2021-02-09

**Authors:** Xinwei Wang, Yaqi An, Pan Xu, Jianwei Xiao

**Affiliations:** ^1^Beijing Advanced Innovation Center for Tree Breeding by Molecular Design, Beijing Forestry University, Beijing, China; ^2^College of Biological Sciences and Biotechnology, Beijing Forestry University, Beijing, China; ^3^State Key Laboratory of Grassland Agro-Ecosystems, College of Pastoral Agriculture Science and Technology, Lanzhou University, Lanzhou, China

**Keywords:** metabolism, gene expression, biogenesis, chloroplast, PPR protein

## Abstract

The pentatricopeptide repeat (PPR) proteins constitute one of the largest nuclear-encoded protein families in higher plants, with over 400 members in most sequenced plant species. The molecular functions of these proteins and their physiological roles during plant growth and development have been widely studied. Generally, there is mounting evidence that PPR proteins are involved in the post-transcriptional regulation of chloroplast and/or mitochondrial genes, including RNA maturation, editing, intron splicing, transcripts’ stabilization, and translation initiation. The cooperative action of RNA metabolism has profound effects on the biogenesis and functioning of both chloroplasts and mitochondria and, consequently, on the photosynthesis, respiration, and development of plants and their environmental responses. In this review, we summarize the latest research on PPR proteins, specifically how they might function in the chloroplast, by documenting their mechanism of molecular function, their corresponding RNA targets, and their specific effects upon chloroplast biogenesis and host organisms.

## Introduction

The family of pentatricopeptide repeat (PPR) proteins is well known because of its abundant members and essential functions in angiosperm species, having been discovered just 20 years ago in a genome sequencing analysis of *Arabidopsis thaliana* (Small and Peeters, 2000). The amino acid composition and structure of PPR proteins are similar to those of tetratricopeptide repeat (TPR) proteins, which usually mediate the interaction among proteins (Rovira and Smith, 2019); both families are encoded by nuclear genes and characterized by a tandem of multiple repeating units. PPR proteins belong to the α-solenoid RNA-binding proteins (RBPs) superfamily, and these RBPs are reported to regulate all steps of the life cycle of messenger RNA (mRNA) (Kramer et al., 2018; Müller-McNicoll et al., 2019). Nonetheless, PPR proteins are also closely related to the family of tandem repeat (TR) proteins, which includes armadillo (ARM), leucine-rich repeats (LRRs), tetratricopeptide, ankyrin (ANK), and WD40 proteins, whose functions have been extensively studied in plants, suggesting a potential role of PPR proteins during stress and development processes (Sharma and Pandey, 2016).

Mutations in PPR-encoding genes are always accompanied by defects in chloroplast biogenesis, pigmentation, and embryo and seed development (Sun et al., 2018; Tadini et al., 2018; Wang X. W. et al., 2020). The corresponding phenotypes affiliated with *PPR* genes disruption probably arise from the loss of one to several mitochondrial or chloroplast gene products that are necessary for organelle development (Barkan and Small, 2014); of course, there are a few exceptions, such as *gun1* and *defectively organized tributaries 4*, which are involved in integrating multiple developmental and stress-related signals in both young seedlings and the leaves of adults (Petricka et al., 2008; Ruckle and Larkin, 2009; Cottage et al., 2010). Nevertheless, dozens of photosynthesis defective mutants can survive during the seedling stage until their seed reserves are depleted (Barkan and Small, 2014). Therefore, the embryo-lethal phenotypes caused by plastid defects may generally be due to a dysfunctional plastid translation system that subsequently prevents the biogenesis of several key factors required for normal chloroplast development and photosynthesis (Zoschke et al., 2016; Tadini et al., 2018). Although there is more evidence to suggest that PPR proteins participate specifically in organellar RNA processing, only a few PPR proteins have been functionally characterized in contrast to the vast majority of PPR members. This discrepancy is mainly attributed to the phenotypes of *PPR* genes disruption being very similar among individual PPR proteins, which makes gene functional studies have more challenges.

### Redefinition and Classification of PPR Proteins’ Motifs

Based on its members’ motif structure, the PPR family can be further classified into two subfamilies, PLS and P. The P-class PPR proteins always contain from 2 to over 30 loosely conserved 35 amino acid PPR (P) motifs (Cheng et al., 2016). In addition, a few P-class subfamily members contain an additional small MutS-related (SMR) domain following an array of P-class PPR motifs, so these are classified as PPR-SMR subgroup (Liu et al., 2013). Actually, similar to SMR, there have been defined several subgroups according to their catalytic C-terminal domains, for example, TGM, TGM CCCH-zinc finger, LAGLIDADG, Mitochondrial RNA polymerase, and PRORP (Manna, 2015). In contrast, the PLS-class subfamily members usually consist of an array of triplets, namely, the canonical P motif, L motif, and S motif; both S and L motifs are related to the PPR motif but with a variable length of 31 amino acids (aa) and 35 or 36 aa, respectively (Barkan and Small, 2014). The PLS subfamily PPR proteins can be further categorized based on the attached domains found downstream of the PPR motifs: E, E+, and DYW, which occur in combination or alone (Cheng et al., 2016; Dedow and Bailey-Serres, 2019).

**FIGURE 1:**
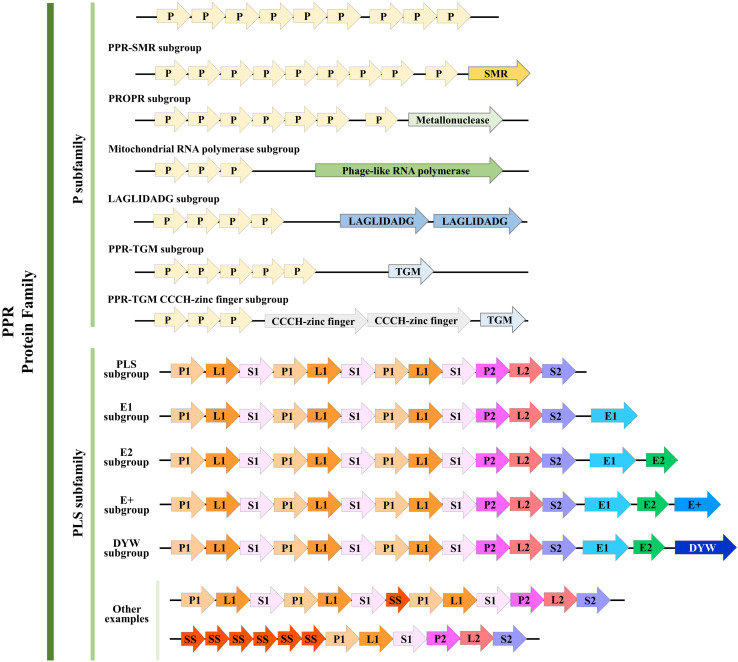
Structure of pentatricopeptide repeat (PPR) proteins. Refined classification and motif structure of PPR proteins (modified from Manna, 2015 and Cheng et al., 2016). The PPR family can be further classified into two subfamilies, P and PLS. P subfamily proteins consist of one or more tandem arrays of P motifs; whereas PLS subfamily proteins consist of triplets of P, L, and S motifs, occasionally interspersed with additional S motifs (P, L, and S can be further divided as we described above). A few P-class proteins contain additional motifs, such as the SMR, TGM, TGM CCCH-zinc finger, LAGLIDADG, Mitochondrial RNA polymerase, and PRORP. Almost all PLS-class proteins contain C-terminal E motifs (E1, E2, and E+; E+ motif was defined by Cheng et al., 2016), and many contain, in addition, a DYW motif. Some other examples that belong to the PLS subfamily were shown here. Arrows with different colors represent different motifs. Numbers of PPR motifs shown in each subgroup do not represent all members, and the motifs are not to scale.

The classification of the PPR proteins family has undergone detailed revision as more species are sequenced and new PPR proteins are discovered. Based on a genomic analysis and comparison of 41 terrestrial plant species with evolutionary differences, the PPR motifs have been redefined as follows (Cheng et al., 2016): the P motif is further divided into P1 and P2, according to a difference in the first helix. Then, in the PLS motif, the L1 and L2 motifs, respectively, consisting of 35 aa and 36 aa can be distinguished, by a difference in the second helix. Similarly, the S motif is also divided into the regular 31-aa S1 motif and the 32-aa S2 motif. Besides the S1 and S2 motifs, a novel S-like motif (31 aa) named SS has been identified that is usually juxtaposed to other S motifs. Meanwhile, the E, E+, and DYW motifs were redefined, and two new 34-aa motifs, E1 and E2, were proposed. In this way, the PLS subfamily was re-divided into six subgroups and a combined P subfamily; here, we summarized the structure and classification of their PPR motifs based on this newly revised definition (Figure 1).

### PPR Family Analysis in Novel Sequenced Species

**FIGURE 2:**
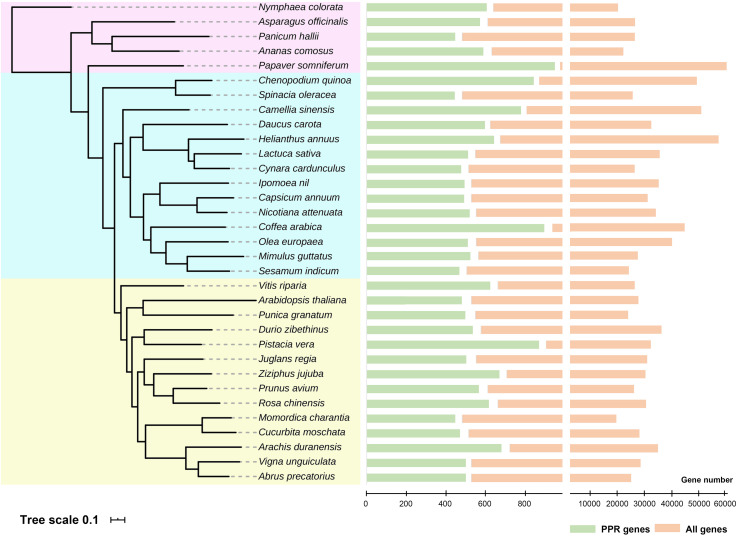
Phylogenetic tree and numbers of pentatricopeptide repeat (PPR) proteins in the entire genomes of recently sequenced plant species. All 33 species were divided into three groups (shown in different background colors), and the numbers of *PPR* genes were shown on the right. Detailed analysis methods were according to Yan et al. (2020). Genome data were obtained from NCBI, and these species were cited as follows: Lurin et al. (2004); Hellsten et al. (2013), Liu et al. (2014); Kim et al. (2014), Wang et al. (2014); Ming et al. (2015), Bertioli et al. (2016); Cruz et al. (2016), Hoshino et al. (2016); Iorizzo et al. (2016), Martínez-García et al. (2016); Scaglione et al. (2016), Badouin et al. (2017); Cui et al. (2017), Harkess et al. (2017); Jarvis et al. (2017), Qin et al. (2017); Reyes-Chin-Wo et al. (2017), Shirasawa et al. (2017); Sun et al. (2017), Teh et al. (2017); Xia et al. (2017), Xu C. et al. (2017), Xu S. et al. (2017), Guo et al. (2018); Lovell et al. (2018), Raymond et al. (2018); Tran et al. (2018), Girollet et al. (2019); Hovde et al. (2019), Lonardi et al. (2019); Zeng et al. (2019), and Zhang L. et al. (2020).

Genomes have been sequenced in various plant species to date. To further reveal the extent of PPR proteins in plants at broader taxonomic scale, we analyzed about 30 species whose entire genomes were sequenced in the last several years. On average, we find about 500 *PPR* genes (filtered pseudo-genes and those alternatively spliced) in each of the various species, but up to 900 members in *Papaver somniferum*. With these data, we then constructed a phylogenetic tree of PPR proteins family in these plants (Figure 2). An outstanding question is this: why do plants have so many PPR proteins yet their bacterial ancestors do not? There are several theories whose adaptive hypotheses are not mutually exclusive, with some support for each of them (Barkan and Small, 2014). To narrow down the possibilities may require more extensive functional studies in PPR proteins and large-scale phylogenetic analysis across a vast number of plant species. However, for now, it is reasonable to conclude that PPR proteins clearly play a critical role in facilitating the chloroplast adaptation to the eukaryotic context (Stern et al., 2010).

## The Function of PPR Proteins in the Chloroplast

Functional chloroplasts are the main sites of photosynthesis, providing both relatively independent space environment and the key proteins for its successful operation, thus constructing the plant’s photoautotrophic condition. Plastid gene expression is indispensable for the development of chloroplasts and their maintenance of normal functions, and PPR proteins can affect it considerably. Thus, it is not surprising that defects in PPR proteins’ functions can yield similar phenotypes associated with chloroplast dysfunction (Manna, 2015). However, there are pronounced differences between the various PPR proteins.

### P-Type PPR Proteins

The P subfamily of PPR proteins is generally believed to take part in RNA stabilization and translational activation and also promote the splicing of group II introns (Barkan and Small, 2014). Firstly, the function of P-class PPR proteins aims at stabilizing specific RNAs in chloroplast. In general, PPR proteins can bind and stabilize the 3′ or 5′ termini of transcription units, also including those termini at the end of a transcript arising from processing between open reading frames (ORFs) in polycistronic units. Moreover, the molecular mechanism depends on diversified PPR motifs bound to a high-affinity RNA target, which can act as a barrier to block exoribonucleases activity. The bound protein determines the actual site of the processed ends, while also preventing the adjacent RNA from undergoing degradation (Barkan and Small, 2014). A classic example, AtBFA2, is necessary for the stabilization of *atpH/F* transcripts in chloroplasts by binding to the *atpF*–*atpA* intergenic region, where it serves as a barrier to protect the *atpH/F* transcripts from destruction by exonucleases (Zhang et al., 2019). Compared with RNA stabilization, subsequent research has shown that translation activation plays a more crucial role in the enhancement of target genes’ expression (Zoschke et al., 2013). It was found that *Zm*PPR10 can bind to the *atpI*–*atpH* intergenic region in the chloroplast, thereby augmenting the efficiency of *atpH* translation. The examples of this function are still obscure, and the underlying mechanism may depend on substrate binding that results in the failed formation of an inhibitory structure by occupying the 5′ end of the hairpin. Meanwhile, blocking this site also reduces the activity of 5′→3′ exoribonucleases, which accordingly stabilized the ORF structure. Thus, RNA stabilization effects and translational enhancement are complementary, as proven repeatedly by studying the function of the LPE1 protein (Prikryl et al., 2011; Williams-Carrier et al., 2019).

P-type PPR proteins feature prominently among a plethora of nucleus-encoded proteins in the splicing of group II introns. Usually, the group II introns in plant organelles have lost the ability of self-splicing and so require other auxiliary factors for their splicing to occur (de Longevialle et al., 2010). It is likely that most of the P-type PPR proteins promote splicing by binding to RNA segments that would otherwise interfere with effective intron folding. Many progresses have been achieved in elucidating such functions in recent years. For example, EMB-7L strongly affects the splicing of multiple plastid or chloroplast transcripts in maize, namely, *trans-rps12*-intron1, *rpl2*, *atpF*, and *ndhA* (Yuan et al., 2019). In our research group, we have characterized two novel PPR factors in *Arabidopsis*: PDM3 and PDM4. In the *pdm3* mutant, intron splicing of *trnA*, *ndhB*, and *clpP-1* was significantly inhibited, and the ensuing phenotypes showed an abnormal chloroplast, inability to engage in photosynthetic autotrophy, and seedling death (Zhang et al., 2017). Concerning the *pdm4* mutant, several group II introns, such as *ndhA*, *petB*, *ycf3-int1*, and *petD*, were also seriously affected, having a similar phenotype to the *pdm3* mutant (Wang X. W. et al., 2020). Unlike multiple targets affected by PDM3 and PDM4, some P-type proteins have been found to act upon a specific target (Ito et al., 2018; Lee et al., 2019; Wang X. M. et al., 2020). Given that plastid intron splicing is a very important process during the gene expression, here we drew a diagram of proteins that facilitate plastid intron splicing (Supplementary Figure 1) based on previous studies (Kroeger et al., 2009; Stern et al., 2010) and the PPR proteins mentioned in this review. Nevertheless, some P subfamily members, for example, ATP4, PDM2, NUWA, and WSL5, are also required for RNA editing of plastid transcripts (Zoschke et al., 2012; Du et al., 2017; He et al., 2017; Liu et al., 2018).

### Mechanism by Which PPR Proteins Recognize an RNA Sequence

Pentatricopeptide repeat proteins are widely considered to be involved in RNA regulation and metabolism in plant organelles. Not surprisingly, the basis for sequence-specific RNA recognition by PPR tracts has been focused upon and studied for a long time. Fortunately, progress on this front has made great strides in understanding of how PPR proteins are able to recognize an RNA target (Barkan et al., 2012; Yin et al., 2013). The theory of the tandem array of PPR motifs is believed to facilitate the recognition and interaction with RNA, and it has been widely accepted in recent years (Yin et al., 2013). By using computational methods, Barkan et al. (2012) inferred a code for nucleotide recognition involving two amino acids in each repeat and then validated their model by designing and recoding a PPR protein to bind novel RNA sequences *in vitro*. These results proved that a PPR protein is capable of binding to an RNA sequence in a parallel orientation *via* a code-recognition mechanism, with nucleotide specificity depending chiefly on the amino acid identities at positions of 6 and 19 in each PPR repeat. In addition, using structural biology analysis, Yin et al. (2013) revealed the molecular mechanism for the specific and modular recognition of RNA bases A, G, and U by the *Zm*PPR10 protein; generally, crystal structures of *Zm*PPR10 were respectively elucidated in either RNA-bound or RNA-free states. In the absence of RNA binding, the 19 PPR repeats of *Zm*PPR10 are folded into a right-handed super-helical spiral. However, in the presence of its targeting ssRNA, PPR10 can assemble into an antiparallel, intertwined homodimer and exhibits substantial conformation changes. Finally, six corresponding PPR10 repeats were found to specifically recognize six nucleotides of *psaJ*, again testifying to the utility of that predicted code (Yin et al., 2013).

### PLS-Type PPR Proteins

Except for the classical P motif, a PLS-type protein always harbors L or S variant PPR motifs and additional C-terminal domains (E, E+, and DYW), which is considered responsible for RNA editing at specific sites (Barkan and Small, 2014). In land plants, RNA editing is a post-transcriptional process that can be fulfilled by deaminating the specific cytidines to uridines, and most of the discovered *trans*-factors involved in this process belong to the PLS subfamily (Shikanai, 2015). It has been proven that the multiple organellar RNA editing factors (MORFs) can interact with the PLS-type PPR proteins and participate in RNA editing. Detailed, the PPR proteins recognize cytidine targets, whereas the MORF proteins modulate the RNA-binding activity of PPR proteins (Huang et al., 2019; Zhao et al., 2019). For instance, MORF9 binding induced significant compressed conformational changes of PPR protein, resulting in that the RNA-binding activity of PPR proteins was drastically increased (Yan et al., 2017). RNA editing can modify the genetic information on RNA molecules and generate translational start or stop codons, which are essential for timely and accurate gene expression (Kotera et al., 2005). In *Arabidopsis* chloroplast, more than 40 of such edited sites have been found (Ruwe et al., 2013), and the machinery and biological function of RNA editing has still not been fully clarified.

The E and DYW motifs are thought to be associated with RNA editing. QED1 and ECD1 in *Arabidopsis* (Wagoner et al., 2015; Jiang et al., 2018) and PPR6 and PPR16 in rice (Tang et al., 2017; Huang et al., 2020), which belong to the PLS-DYW subclass, all affect the RNA editing of different sites, and they can show varied levels of mutational phenotypes. Focusing here on PPR6, its corresponding *ppr6* mutant showed early defective chloroplast development that led to albino leaves and eventual seedling death. In further comparing the sequences of 19 edited sites in the rice chloroplast, only the *ndhB* transcript differs between *osppr6* mutants and wild-type plants. Compared with the wild-type, this cytidine was only edited to uridine about 30%, indicating that the *osppr6* mutant still harbored 30% of its RNA editing activity. Therefore, OsPPR6 specifically edits the transcript of chloroplast *ndhB*, and the reduced editing efficiency of this site in *osppr6* probably caused the disrupted function of the NADH dehydrogenase subunit 2 (Tang et al., 2017). A PSL-E member, PPR756 in rice, was proven to participate in editing events among three mitochondrial genes, rather than in the chloroplast *per se* (Zhang Q. et al., 2020). This finding further confirms that PLS-type PPR proteins typically act as site-specific editing factors during RNA editing in plant organelles. Interestingly, similar to exceptions of P-type PPR proteins, there are also some special PLS PPR proteins: OsWSL, OsSLA4, and PGL12, which are responsible for RNA splicing (Tan et al., 2014; Wang et al., 2018; Chen et al., 2019).

### PPR Protein Directly or Indirectly Involved in the Regulation of Chloroplast Development

Chloroplast biogenesis is highly complex, and the intricate molecular mechanisms have not been fully characterized yet. However, the complexity of this process is understandable because of its ancestry, having originated *via* endosymbiosis with species of cyanobacteria (Cortleven and Schmülling, 2015). With its own separate genome, signaling connections are heavily relied upon to relay information between the nucleus and chloroplast genomes, and the development of functional and photosynthetically active chloroplasts depends on the proper formation and assembly of molecules (Pogson et al., 2015). Corroborating this, it has been demonstrated that PPR proteins participate in chloroplast gene expression and function (Barkan and Small, 2014).

Based on the above, we may summarize that various PPR proteins can foster a similar phenotype with respect to chloroplast, such as pale-yellowish pigmentation, altered PSII biogenesis and formation of grana thylakoid, hindered chlorophyll synthesis, and severe defects in photosynthesis. Upon further analysis, PPR proteins can directly or indirectly affect chloroplast biogenesis or development processes through a variety of action modes and functional types, which can lead to abnormal chloroplast development, or almost an entire loss of functioning chloroplast, leading to the death of embryos or seedlings. According to our reviewed material, we summarized the known PPR proteins’ respective activity in recent years in Supplementary Table 1 and drew a simple working model to further summarize the function of PPR proteins (Supplementary Figure 2).

## Conclusion and Perspectives

In general, PPR proteins are divided into the PLS and P subfamilies, and they are usually involved in RNA editing or RNA stabilization, cleavage, and splicing. However, with the deepening and accumulating research, not all PPR proteins conform to this law. Maybe plenty of evolutionary analyses and crystal structures of PPR–RNA complexes will provide deeper insight into subtle classification and functional mechanism. Besides, more attention should be paid to PPR interacting proteins that could severely affect the PPR protein effects, such as MORFs and/or RNA editing factor interaction proteins (RIPs) (Zhao et al., 2019). Excitingly, some of new research studies suggest that artificial PPR proteins could be customized to bind specific endogenous RNA *in vivo*, thus providing infusive research prospects for the development of designer RBPs for applications in biotechnology and synthetic biology (McDermott et al., 2019).

## Author Contributions

JX designed the outlines in this review and edited the manuscript. XW and YA wrote the original manuscript. PX analyzed the genomic data. All authors read and approved the final manuscript.

## Conflict of Interest

The authors declare that the research was conducted in the absence of any commercial or financial relationships that could be construed as a potential conflict of interest.

## Supplementary Material

Supplementary Figure 1 Proteins that facilitate plastid intron splicing. Introns are designated as subgroup I, IIA, and IIB, according to Michel et al. (1989). The oval represents the PPR proteins; quadrilateral refers to those proteins belonging to other families. Solid lines indicate the recent research progress mentioned in our present review paper; dashed lines indicate previous results. The arrows point to their targets.

Supplementary Figure 2 Simple working models of PPR proteins. PPR proteins can interact with RNA to affect gene expression by several ways (modified and refined from Barkan and Small, 2014). **(A)** PPR protein could expose (or mask) *cis*-elements for RNA translation, RNA splicing, RNA cleavage, or RNA editing. **(B)** PPR protein can activate translation via preventing the formation of an RNA structure, which masks the ribosome-binding site. **(C)** PPR protein can stabilize RNAs by blocking 5′→3′ and 3′→5′ exoribonucleases. Different PPR proteins are shown in different colors.

Supplementary Table 1 Phenotypes and functions of PPR proteins in plant growth and development according to this review were summarized in recent years (since 2015). Location: C, chloroplast; M, mitochondrion.
