# Supplementary figures and images for: Functioning of PPR Proteins in Organelle RNA Metabolism and Chloroplast Biogenesis
Source: Front Plant Sci. 2021 Feb 9;12:627501. doi: 10.3389/fpls.2021.627501 (PMC7900629; doi:10.3389/fpls.2021.627501)

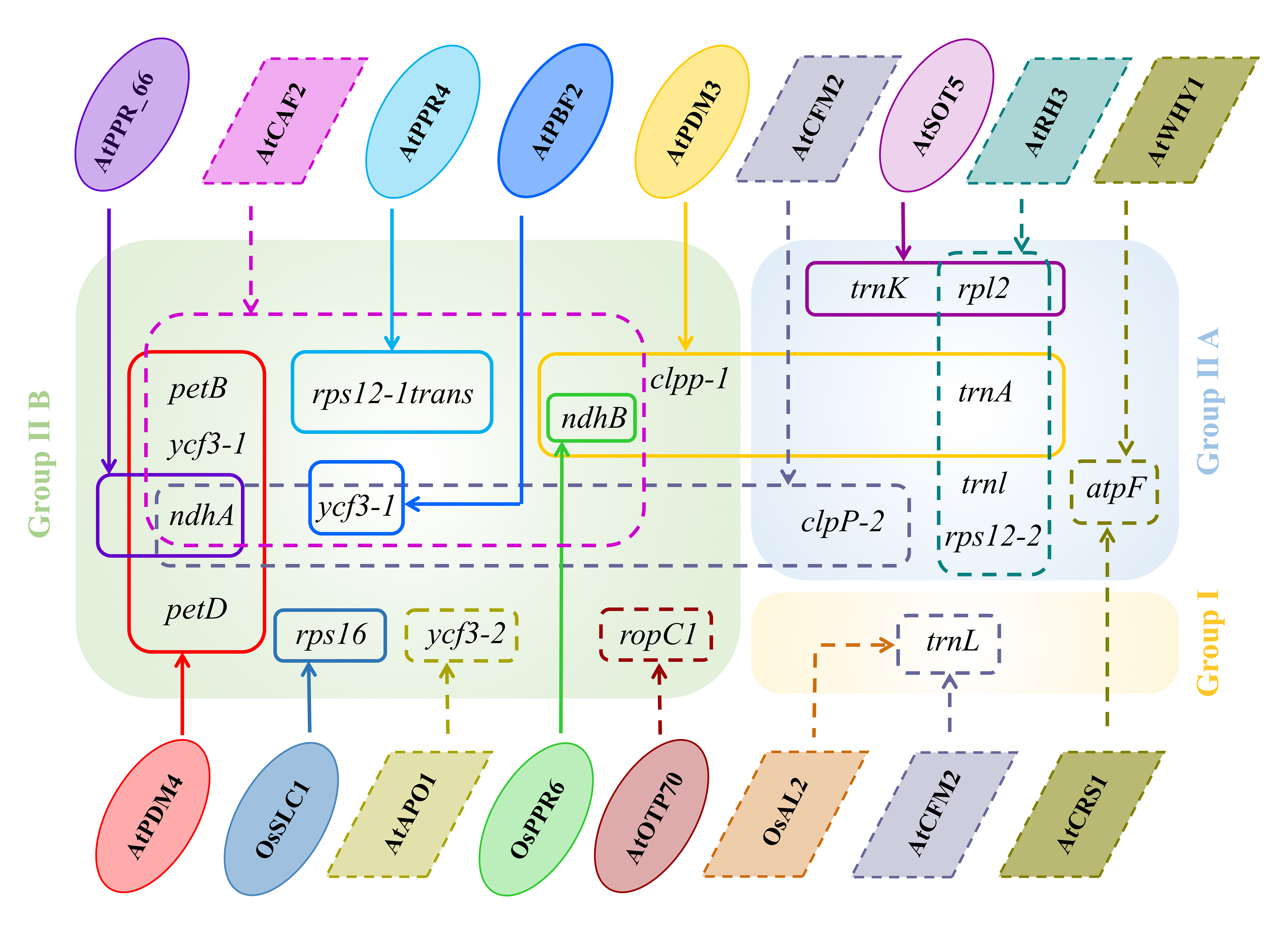

Supplement: Supplementary Figure 1 — Proteins that facilitate plastid intron splicing. Introns are designated as subgroup I, IIA, and IIB, according to Michel et al. (1989). The oval represents the PPR proteins; quadrilateral refers to those proteins belonging to other families. Solid lines indicate the recent research progress mentioned in our present review paper; dashed lines indicate previous results. The arrows point to their targets. [file Image_1.JPEG]

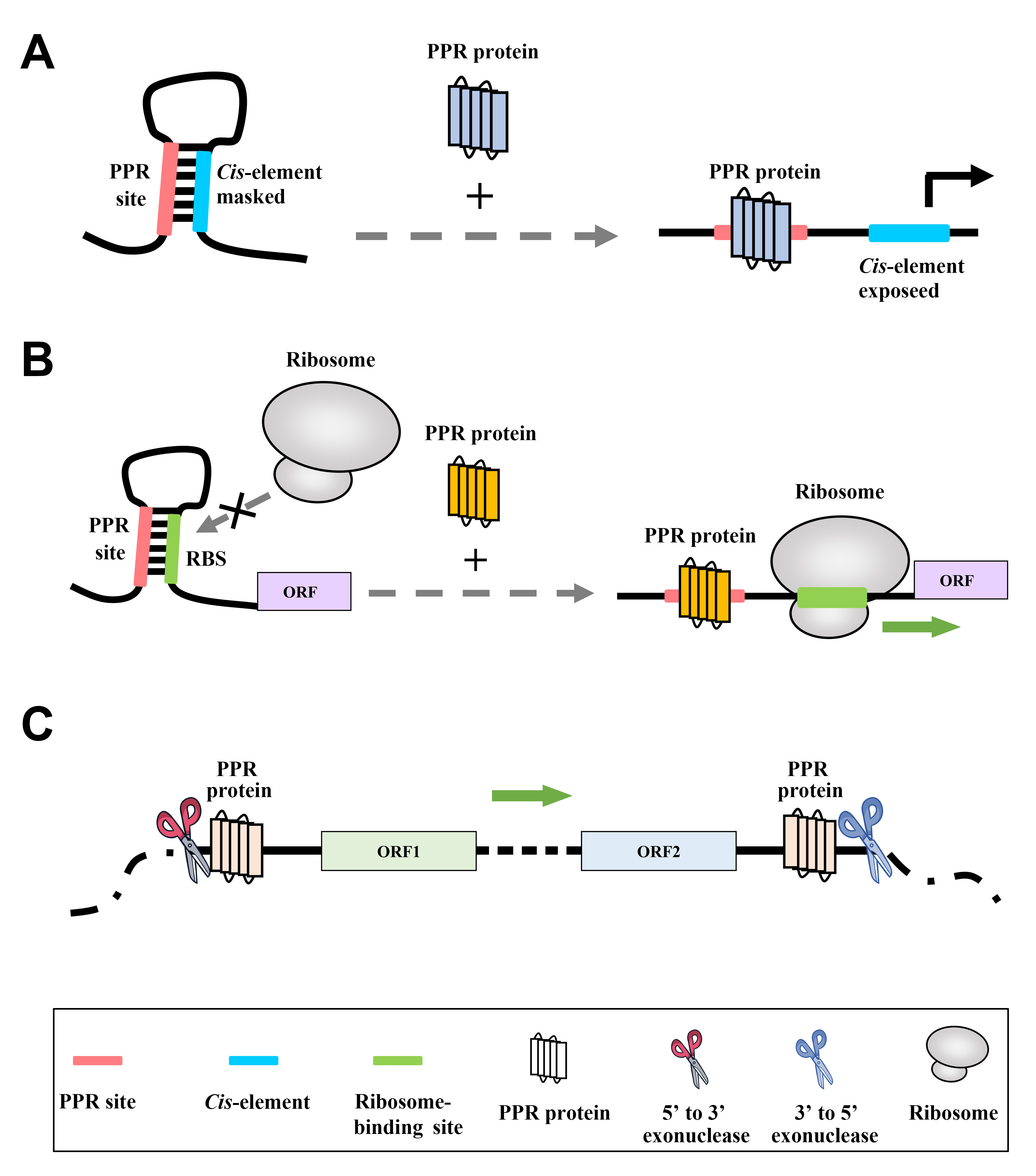

Supplement: Supplementary Figure 2 — Simple working models of PPR proteins. PPR proteins can interact with RNA to affect gene expression by several ways (modified and refined from Barkan and Small, 2014). (A) PPR protein could expose (or mask) cis-elements for RNA translation, RNA splicing, RNA cleavage, or RNA editing. (B) PPR protein can activate translation via preventing the formation of an RNA structure, which masks the ribosome-binding site. (C) PPR protein can stabilize RNAs by blocking 5′→3′ and 3′→5′ exoribonucleases. Different PPR proteins are shown in different colors. [file Image_2.JPEG]
