# Supplementary material for: Functioning of PPR Proteins in Organelle RNA Metabolism and Chloroplast Biogenesis
Source: Front Plant Sci. 2021 Feb 9;12:627501. doi: 10.3389/fpls.2021.627501 (PMC7900629; doi:10.3389/fpls.2021.627501)
Supplement: Supplementary Table 1 — Phenotypes and functions of PPR proteins in plant growth and development according to this review were summarized in recent years (since 2015). Location: C, chloroplast; M, mitochondrion. [file Table_1.docx]

| **Plant** | **PPR protein**  **(year published)** | **Location** | **Molecular function** | **Mutant phenotype** | **Ref.** |
| --- | --- | --- | --- | --- | --- |
| ***A. thaliana*** | SLO3 P-type  (2015) | M | Splicing of the NADH dehydrogenase subunit7 (*nad7*) intron 2 | Significant reduction in the activity of mitochondrial complex I | Hsieh et al., 2015 |
|  | BLX PLS-DYW  (2018) | M | Editing in mitochondria;  splicing the fourth intron of *nad1* and the first intron of *nad2* | Embryo lethality, and the endosperm failed to initiate cellularization | Sun et al., 2018 |
|  | MID1 P-type (2020) | M | Splicing of the *nad2* intron 1 | Embryo development and stunted plant growth, with defects in cell expansion and proliferation | Zhao et al., 2020 |
|  | NUWA P-typr  (2017) | M | The mechanism of how NUWA imprinting regulated is not yet clear | Defects in early embryogenesis and endosperm development | He et al., 2017 |
|  | PDM2 P-type  (2017) | C | Affecting plastid  RNA editing efficiency in most editing sites | Lethal phenotype accompanied by albino cotyledons | Du et al., 2017 |
|  | PDM3 P-type (2017) | C | Splicing of *trnA*, *ndhB*, and *clpP-1* | Abnormal chloroplast; seedling death | Zhang et al., 2017 |
|  | PDM4 P-type (2020) | C | Splicing of the group II intron of *ndhA*, *petB*, *ycf3-int-1*, *petD* | Similar to *pdm3* | Wang X. W. et al., 2020 |
|  | PPR4 P-type  (2018) | C | *Trans*-splicing of *rps12* | Lethal to embryos | Tadini et al., 2018 |
|  | PBF2 P-type  (2020) | C | Specific to the intron splicing of *ycf3* | Lethal to seedlings; pale and yellowish; smaller, developed more slowly | Wang X. M. et al., 2020 |
|  | PPR_66 P-type (2018) | C | Splicing of *ndhA* transcript | No visible phenotype | Ito et al., 2018 |
|  | BFA2 P-type (2019) | C | Stabilization of *atpH/F* | Stunted growth | Zhang et al., 2019 |
|  | LPE1 P-type  (2019) | C | Promotion of PSII  synthesis by activating translation of the *psbJ* ORF | Affects PSII biogenesis and the formation of grana thylakoids in the chloroplast | Williams-Carrier et al., 2019 |
|  | QED1 PLS-DYW (2015) | C | Affects five different plastid-editing sites | Stunted growth and accumulation of apparent photodamage | Wagoner et al., 2015 |
|  | ECD1 PLS-DYW (2018) | C | RNA editing of *rps14-149* | Embryo lethality | Jiang et al., 2018 |
| ***Z. mays*** | PPR14 P-type (2020) | M | Splicing of *nad2* and *nad7* | Abnormal endosperm development, and impaired NADH dehydrogenase activity | Wang H et al., 2020 |
|  | EMB-7L P-type  (2019) | C | Affects the splicing of multiple chloroplast transcripts | Embryo lethality and albinism | Yuan et al., 2019 |
|  | PPR4 P-type  (2019) | C | *Trans*-splicing of *rps12* | Seedling‑lethal phenotype | Lee et al., 2019 |
|  | PPR53 P-SMR  (2016) | C | Stabilization and translation enhancing  effects at the *ndhA* locus | Chlorosis, lethal to seedlings | Zoschke et al., 2016 |
| ***O. sativa*** | SLC1 P-type  (2020) | C | Mainly influences the splicing of *rps16* and multiple group II introns | Low contents of  photosynthetic pigments in leaves; abnormal chloroplast development; severely defective photosynthesis | Lv et al., 2020 |
|  | SLA4 PLS-DYW  (2018) | C | Influencing the intron  splicing of multiple chloroplast group II introns | Seedling‑lethal albino  phenotype | Wang et al., 2018 |
|  | WSL5 P-type  (2018) | C | RNA editing of *rpl2* and *atpA*; splicing of *rpl2* and *rps12* | White-striped leaves during early leaf development and is albinic when planted under cold stress | Liu et al., 2018 |
|  | PGL12 PLS  (2019) | C | 16S rRNA process-  ing and splicing of the plastid transcript *ndhA* | Yellow-green leaves at seedling stage and turned pale green as the plants grew | Chen et al., 2019 |
|  | PPR756 PLS-E (2020) | C/M | RNA editing of *apt6*, *ccmC*, *nad7* | Growth retardation in early development; pollen sterility at the reproductive stage | Zhang et al., 2020 |
|  | PPR6 PLS-DYW (2017) | C | Editing of *ndhB* and splicing of *ycf3* transcripts | Early chloroplast developmental defects; albino leaves and seedling death | Tang et al., 2017 |
|  | PPR16 PLS-DYW (2020) | C | Responsible for a single editing event in the chloroplast *rpoB* mRNA | Chlorophyll synthesis and chloroplast development are hindered | Huang et al., 2020 |
